# Daptomycin-Loaded Nanocarriers Facilitate Synergistic Killing of Methicillin-Resistant *Staphylococcus aureus* via Lipid-Mediated Interactions and Targeting

**DOI:** 10.1093/infdis/jiaf492

**Published:** 2025-11-04

**Authors:** Jhih-Hang Jiang, Chia Xin Lim, Xiangfeng Lai, Xenia Kostoulias, Faye C Morris, Anton P Le Brun, Chun-Ming Wu, Nageshwar R Yepuri, Hsin-Hui Shen, Anton Y Peleg

**Affiliations:** Infection Program, Monash Biomedicine Discovery Institute and Department of Microbiology, Monash University, Clayton, Victoria, Australia; Department of Infectious Diseases, The Alfred Hospital and School of Translational Medicine, Monash University, Melbourne, Victoria, Australia; Centre to Impact AMR, Monash University, Clayton, Victoria, Australia; Biomedicine Discovery Institute and Department of Biochemistry, Monash University, Clayton, Victoria, Australia; Department of Materials Science and Engineering, Faculty of Engineering, Monash University, Clayton, Victoria, Australia; Infection Program, Monash Biomedicine Discovery Institute and Department of Microbiology, Monash University, Clayton, Victoria, Australia; Department of Infectious Diseases, The Alfred Hospital and School of Translational Medicine, Monash University, Melbourne, Victoria, Australia; Centre to Impact AMR, Monash University, Clayton, Victoria, Australia; Infection Program, Monash Biomedicine Discovery Institute and Department of Microbiology, Monash University, Clayton, Victoria, Australia; Centre to Impact AMR, Monash University, Clayton, Victoria, Australia; Australian Centre for Neutron Scattering, Australian Nuclear Science and Technology Organisation, Kirrawee DC, New South Wales, Australia; National Synchrotron Radiation Research Center, Hsinchu Science Park, Hsinchu, Taiwan; National Deuteration Facility, Australian Nuclear Science and Technology Organisation, Kirrawee DC, New South Wales, Australia; Biomedicine Discovery Institute and Department of Biochemistry, Monash University, Clayton, Victoria, Australia; Department of Materials Science and Engineering, Faculty of Engineering, Monash University, Clayton, Victoria, Australia; Infection Program, Monash Biomedicine Discovery Institute and Department of Microbiology, Monash University, Clayton, Victoria, Australia; Department of Infectious Diseases, The Alfred Hospital and School of Translational Medicine, Monash University, Melbourne, Victoria, Australia; Centre to Impact AMR, Monash University, Clayton, Victoria, Australia

**Keywords:** cubosomes, MRSA, daptomycin, cell membrane, antimicrobial resistance

## Abstract

Preservation and augmentation of existing antimicrobials is crucial in combating antimicrobial resistance. Gram-positive bacteria, exemplified by *Staphylococcus aureus*, are among the most common human bacterial pathogens, with methicillin-resistant *S. aureus* (MRSA) now established globally. Daptomycin is a last-line anti-staphylococcal antimicrobial that uniquely targets the bacterial membrane with bactericidal effects. Here, we developed lipid-based nanoparticles, namely cubosomes, to encapsulate daptomycin for targeted delivery via lipid-mediated interactions. Daptomycin-loaded cubosomes synergistically killed 14 clinical MRSA isolates in vitro compared with daptomycin or cubosomes alone. This synergy between daptomycin and cubosome was mediated by cubosomes docking on the *S*. *aureus* cell surface, releasing daptomycin for membrane extraction and penetration, followed by lipid cubosome infusion into *S*. *aureus* membranes. Using a murine septicemia model, daptomycin-loaded cubosomes significantly reduced the organ bacterial burden of MRSA. Together, these data showed that multifunctional lipid nanocarriers can potentiate the bactericidal activity of daptomycin using a membrane-targeted trojan-horse-like mechanism.

## Teaser

Encapsulation of daptomycin in lipid-based nanoparticles, namely cubosomes, shows synergistic killing of methicillin-resistant *Staphylococcus aureus* in vitro and in vivo. This synergy is mediated by specific and combined bacterial membrane targeting mechanisms. This study highlights a potential innovative therapeutic strategy.

Cell membranes are crucial for bacterial survival, as these semipermeable barriers allow for metabolite transport, energy production, and cell division [1]. In gram-positive bacteria, the membranes typically contain significant amounts of phosphatidylglycerols and cardiolipins [2], which are rarely found in eukaryotic cell membranes, making bacterial cell membranes ideal targets for antimicrobials. Daptomycin is the only cyclic lipopeptide antimicrobial approved for clinical use to treat a range of gram-positive bacterial infections, including methicillin-resistant *Staphylococcus aureus* (MRSA) and *Enterococcus* species [3]. It is a last-line, bactericidal antimicrobial that is approved for the treatment of complicated skin and soft tissue infections, bacteremia, and right-sided infective endocarditis caused by *S*. *aureus* [3]. It targets the bacterial cell membrane in a calcium-dependent manner [3], but the mechanisms of bacterial killing are likely multifactorial and bacterial species dependent [4]. Studies have shown that daptomycin triggers membrane depolarization, loss of intracellular components, and inhibition of cell envelope synthesis [5–7]. Unfortunately, there have been increasing reports of resistance emerging to daptomycin associated with clinical failure, often in the setting of complicated, deep-seated infections such as osteomyelitis, septic arthritis, left-sided infective endocarditis, and prosthetic joint infections [8–10]. To preserve this important last-line antimicrobial, novel strategies are required to potentiate the bacterial membrane–targeting effects of daptomycin for improved treatment outcomes.

Cubosomes are lipid nanoparticles that have been shown to be effective biocompatible carriers for drugs and peptides for therapeutic delivery to treat cancer, burns, and rheumatoid arthritis [11]. They are made from lipidic cubic–phase structures that have high membrane curvature and surface area [11, 12] and can facilitate carriage of a broad range of therapeutic molecules, including hydrophilic, hydrophobic, and amphiphilic substances [11]. Cubosomes can also provide protection of the loaded drug [13], enhance penetration into deep tissues [14], and have an increased surface area to encapsulate higher amounts of drug compared with liposomes [15]. Notably, the lipid composition of cubosomes can be tailored to interact with eukaryotic or bacterial membranes, allowing specialized targeting with the potential for reduced toxicity [16, 17]. Phytantriol is a structurally stable aliphatic alcohol and is a commonly used lipid to form cubosomes. We and others have shown that phytantriol-based cubosomes can be engineered to interact and incorporate into lipid membranes [18, 19].

In this study, we exploited the bacterial membrane targeting of daptomycin with the lipid exchange properties of phytantriol-based cubosomes, to create a smart lipid nanocarrier that can function via a trojan-horse-like antimicrobial delivery mechanism. After developing and characterizing our daptomycin-loaded cubosomes, we showed the efficacy of daptomycin-loaded cubosomes in eradicating MRSA cells using in vitro treatment assays and a murine staphylococcal bacteremia model. Through super-resolution microscopy and neutron reflectometry (NR), we uncovered the bactericidal mechanisms of daptomycin-loaded cubosomes interacting with staphylococcal membranes, providing the necessary insights into a highly specific synergy between a membrane targeting antimicrobial and a uniquely tailored, lipid-based delivery system.

## METHODS

### Methicillin-Resistant *Staphylococcus aureus* Strains, Antibacterial Susceptibility Testing, and Time-Kill Assays

Clinical MRSA isolates (Table 1) were tested for antibacterial susceptibility using the broth microdilution following the Clinical and Laboratory Standards Institute guidelines. Overnight cultures in cation-adjusted Mueller–Hinton broth (MHB) were diluted to 10^6^ CFU/mL in MHB with 50 mg/L Ca^2+^ (CaMHB). In 96-well plates, 50 μL of bacterial suspension was mixed with 50 μL of antibiotics or daptomycin-loaded cubosomes. After 16–20 hours incubation, minimum inhibitory concentrations were recorded. Minimum bactericidal concentrations (MBCs) were determined by plating on brain heart infusion (BHI) agar. All experiments were done in biological triplicates. For time-kill assays, MRSA cultures were diluted to 10^6^ CFU/mL in 5 mL CaMHB and treated accordingly. Viable cells were quantified by plating on BHI agar at 0, 4, 8, 12, and 24 hours.

**Table 1.** Clinical MRSA Isolates and MBCs of Free Daptomycin or Daptomycin in Daptomycin-loaded Cubosomes (Dp-Cub)

Strains (CC Type)^a^	Daptomycin MIC (µg/mL)	Daptomycin MBC (µg/mL)	Daptomycin MBC (µg/mL) in Dp-Cub	Reference
A8819 (5)	0.25	2	0.5	[8]
A9754 (8)	0.5	4	1	[8]
A9763 (5)	0.5	2	0.5	[9]
A10102 (5)	0.5	2	0.5	[10]
A9299 (5)	0.25	4	2	[8]
A9719 (5)	0.25	16	4	[8]
A8796 (5)	0.5	16	4	[20]
A9765 (8)	0.5	16	2	[9]
A9781 (5)	0.5	4	1	[8]
A8090 (5)	0.25	16	1	[21]
A5937 (5)	0.125	8	2	[22]
A5940 (5)	0.25	8	2	[22]
A6224 (5)	0.25	4	1	[23]
A6300 (5)	0.25	8	2	[22]

Abbreviations: MRSA, methicillin-resistant *Staphylococcus aureus*; MIC, minimum inhibitory concentrations; MBC, minimum bactericidal concentration.

^a^CC, clonal complex based on multilocus sequence typing.

### Synthesis of Cubosomes

Unloaded cubosomes were prepared by melting 2.3 wt% 1,2-dihexadecanoyl-sn-glycero-3-phospho-L-serine (DPPS), 88.6 wt% 1,2,3-trihydroxy-3,7,11,15-tetramethylhexadecane (phytantriol), 9.1 wt% Pluronic F-127, and ultrapure water at 70°C to form a cubic-phase gel, then dispersing it in saline via probe sonication [18]. For daptomycin-loaded cubosomes, 4.5–22.5 wt% daptomycin was added to gels with varying compositions DPPS, F-127, and phytantriol. Fluorescent cubosomes were produced by adding octadecyl rhodamine B chloride to phytantriol at a 1:2000 weight ratio. Hydrodynamic diameter and polydispersity index were measured by dynamic light scattering (DLS) on a Zetasizer Nano ZS (Malvern, United Kingdom). Deuterated cubosomes were made using phytantriol-d_39_ (Australian Nuclear Science and Technology Organisation) [24].

### Cryo-transmission Electron Microscopy

Cubosomes were applied to carbon-coated copper grids and vitrified in liquid ethane. Samples were imaged using a Tecnai 12 Cryo-Transmission Electron Microscope (Cryo-TEM; FEI, The Netherlands) with a Gatan 626 cryo holder at 120 kV and 8–10 electrons/Å^2^. Images were collected with a Megaview III CCD camera using AnalySIS software (Olympus; Supplementary Methods).

### In Vitro Release of Daptomycin from Daptomycin-Loaded Cubosomes

Daptomycin release from loaded cubosomes was assessed using dynamic dialysis. A 0.5 mL (2.5 mg/mL daptomycin in cubosomes or free drug) was sealed in a 10 000 MWCO dialysis bag and immersed in 25 mL phosphate-buffered saline (PBS, pH 7.4) at 37°C with shaking (200 rpm). At each time point, 1 mL was sampled and replaced with fresh PBS. Daptomycin concentration was quantified by high-performance liquid chromatography (HPLC) [25] using a Waters Spherisorb 136 ODS-2 reversed-phase C18 column, 1 mL/min flow rate, and a mobile phase of 30% acetonitrile/70% 0.05 M phosphate buffer (pH 5.5). Detection was at 223 nm. Experiments were done in technical triplicates.

### Murine Infection Model

*Staphylococcus aureus* A8819 was cultured overnight in BHI, centrifuged, and resuspended in PBS. Approximately 8 × 10^7^ CFU in 0.1 mL PBS was injected into 6-week-old female BALB/c mice via the tail vein (5 mice per group). One-hour postinfection, mice received intraperitoneal treatments: (1) saline (control), (2) cubosomes (45.6 mg/kg), (3) daptomycin (10 mg/kg), or (4) 18.0 wt% daptomycin-loaded cubosomes (10 mg/kg daptomycin, 45.6 mg/kg cubosomes). Mice were monitored ≥3 times in 24 hours, with humane euthanasia by CO_2_ if distressed. After 24 hours, kidneys, livers, and spleens were collected, homogenized, serially diluted, and plated on BHI agar to quantify viable bacteria after 16 hours incubation at 37°C. All procedures were approved by the Monash University Animal Research Platform Ethics Committee (E/1781/2018/M).

### Neutron Reflectivity of *Staphylococcus aureus* Bilayer Membrane

*Staphylococcus aureus* membrane bilayers were formed on silicon oxide (SiO_2_) surfaces using published methods [26, 27]. Specular neutron reflectivity was measured after 4 hours daptomycin-packaged cubosomes treatment using the Platypus and Spatz time-of-flight reflectometer (2.8–18 Å spectrum range) at the OPAL reactor (Australia) [26, 28, 29]. Data were collected on a 2D ^3^He detector with (60 mm footprint) at 0.85° and 3.5° incident angles in 100% D_2_O, 100% H_2_O, and contrast match silicon (CmSi, 62% H_2_O/38% D_2_O). Data were reduced using refnx [30], and reflectivity was plotted as a function of momentum transfer, *Q* = *4π* sin *θ/λ*.

NR analysis was carried out using the Refnx MOTOFIT [31]. The bilayer was modeled as 3 slabs: inner leaflet headgroups, tails, and outer leaflet headgroups. Slabs under different isotopic contrasts (D_2_O, H_2_O, and CmSi) were fitted simultaneously to minimize χ^2^ using a differential evolution algorithm. The best-fit model provided theoretical neutron scattering length densities (SLDs, 10^−6^ Å^−2^), thickness and roughness of each slab. For slabs with 2 components (species *s* and water *w*), SLD was calculated as:


$$P_{\mathrm{layer}}=\phi P_{s}+(1-\phi )P_{w}$$

where *ϕ* is the volume fraction of species s. *P_w_* and *P_s_* are the SLDs of the 2 components. Parameters uncertainties were estimated using a Bayesian Markov Chain Monte Carlo approach on the best-fit models [32]. Frequency plot of fitted values were analyzed, and errors were defined as twice the standard deviation (SD), with the parameter values taken as the midpoint of the 95% confidence interval (Supplementary Methods).

### Super-Resolution Microscopy

*Staphylococcus aureus* cells were stained with Wheat Germ Agglutinin-Alexa Fluor™ 488, washed by centrifugation, and incubated with RhD-B-labeled daptomycin-packaged cubosomes. After incubation, 10 µL of the suspension was placed on poly-L-lysine-coated coverslips. Live-cell imaging was performed using a Zeiss LSM 980 Airyscan 2 microscope (32 + 2-channel GaAsP detector and Airyscan 2 detectors) with a 63× oil C PlanApo 1.4NA objective. The images were processed in ImageJ.

### Statistical Analysis

Data are presented as mean ± SD. Statistical analyses and area under the curve calculations for time-kill assays were performed using GraphPad Prism 10.0. Statistical tests were detailed in figure legends; *P* < .05 was considered significant.

## RESULTS

### Development and Characterization of Daptomycin-Loaded Cubosomes

**Figure 1.**
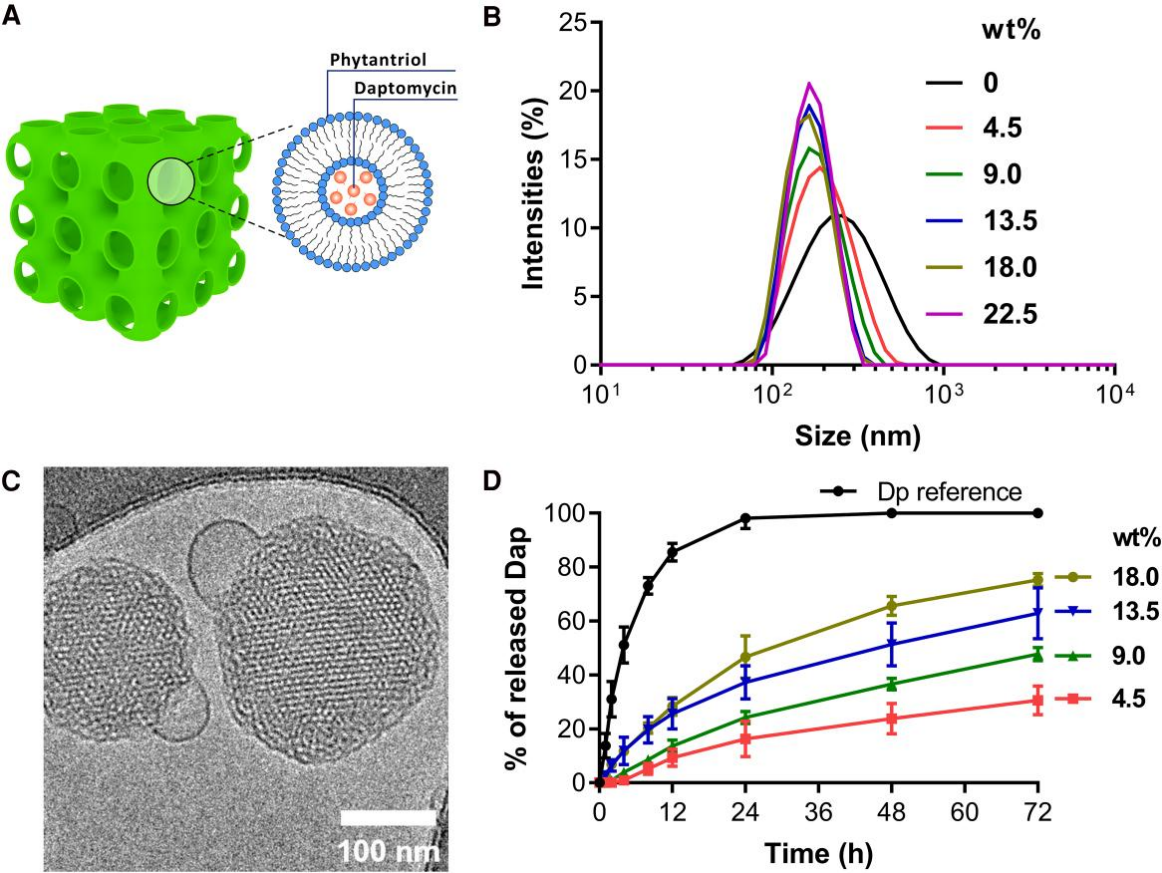
Characterization of daptomycin-loaded cubosomes. *A*, Schematic illustration of cubosomes loaded with daptomycin. *B*, Dynamic light scattering profiles of daptomycin-loaded cubosomes. *C*, Representative cryo-TEM image of 18.0 wt% daptomycin-loaded cubosomes. The scale bar is 100 nm. *D*, Release of daptomycin from daptomycin-loaded cubosomes was measured by high-performance liquid chromatography. Reference with pure daptomycin or daptomycin-loaded cubosomes was dialyzed in PBS. Error bars indicate mean ± SD of triplicates from 1 representative experiment.

Cubic nanostructures are formed through the physico-chemical properties of phytantriol [33], with the loaded antimicrobial being packaged within protected membrane compartments (Figure 1*A*). We first packaged daptomycin in phytantriol-based cubosomes at varying concentrations (mass fraction 4.5, 9.0, 13.5, 18.0, and 22.5 wt%), and using DLS, showed that the diameters of the daptomycin-loaded cubosomes were 150–300 nm (Figure 1*B*, Supplementary Table 1). Using a measure of size variation, known as the polydispersity index, we showed that there was a homogenous population of nanoparticles (values below 0.3) (Supplementary Table 1) [34]. The internal structure of the packaged cubosomes was also characterized using small-angle X-ray scattering (SAXS), showing that daptomycin mass fractions between 4.5–18 wt% allowed appropriate cubic structures to form, whereas excess loading (22.5 wt%) led to the formation of disordered structures (Supplementary Table 1, Supplementary Figure 1) [35]. Cryogenic transmission electron microscopy (Cryo-TEM) images validated that 4.5–18.0 wt% daptomycin-loaded cubosomes possessed similar ordered structures of cubic phase when compared with unloaded cubosomes (Figure 1*C*, Supplementary Figure 2). We also quantified the kinetics of daptomycin release from our packaged cubosomes (Figure 1*D*). Slower and more sustained release was observed compared with free daptomycin, and it was correlated with the amount of daptomycin loading (Figure 1*D*) and the internal structure of the cubosomes. The 18.0 wt% daptomycin-loaded cubosomes exhibited the highest release rate, and this was due to a reduction of the bilayer curvature within the cubosomes (Supplementary Figure 1), whilst 4.5 wt% daptomycin-loaded cubosomes had the lowest release rate (Figure 1*D*). These data led us to select 18.0 wt% daptomycin-loaded cubosomes for ongoing investigation.

### Enhanced Bactericidal Activities of Daptomycin-packaged Cubosomes

**Figure 2.**
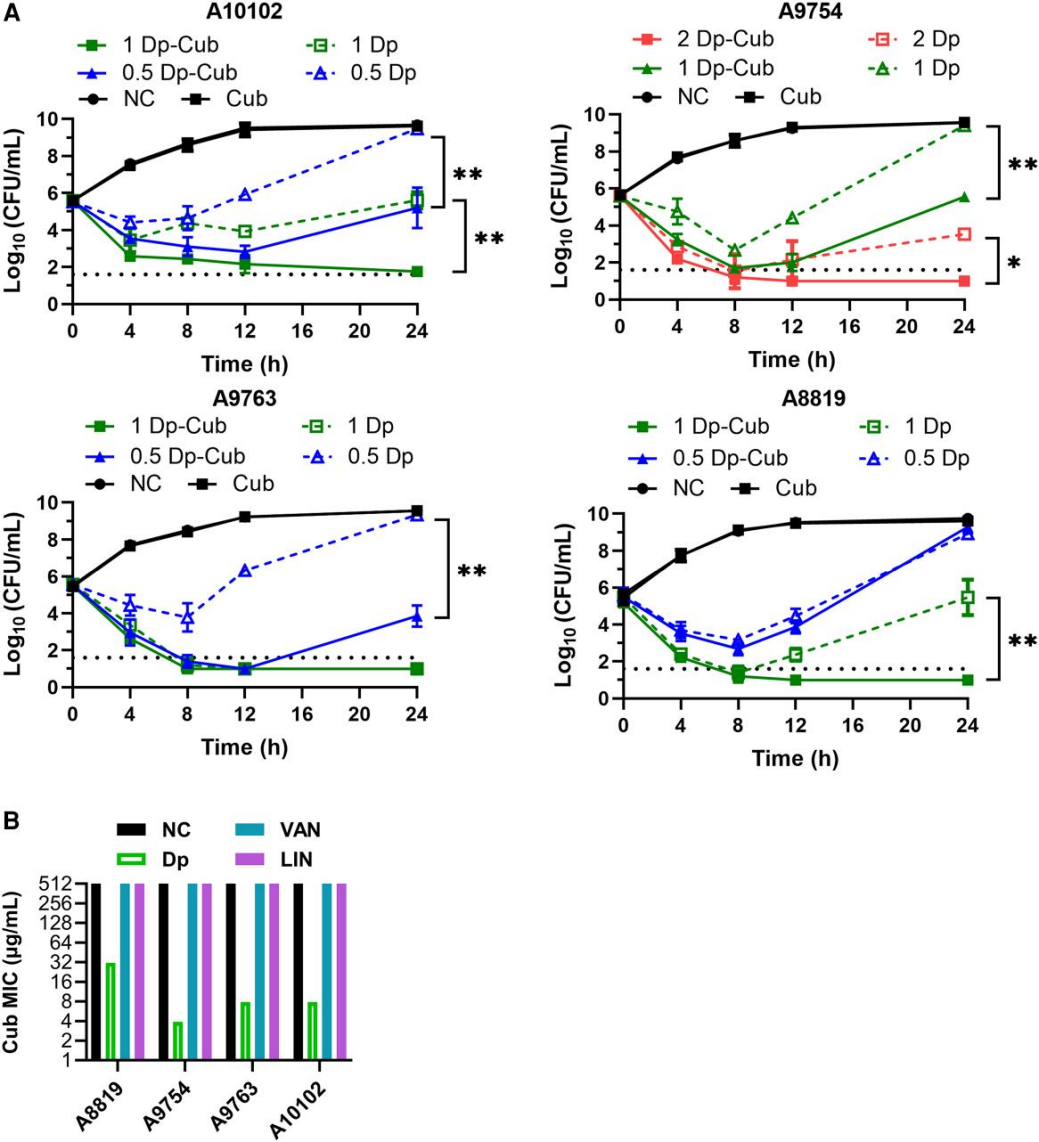
In vitro antimicrobial activities of daptomycin-loaded cubosomes against clinical MRSA isolates. *A*, Time-kill analyses of viable bacterial counts for A10102, A9754, A9763, and A8819 during treatments are shown. The cells were exposed to 0.5, 1, and 2 μg/mL daptomycin (Dp), equivalent doses of daptomycin (0.5, 1, and 2 μg/mL) packaged in cubosomes (Dp-Cub), cubosomes 31 μg/mL (Cub), or saline (negative control, NC). Dot line indicates the detection limit. Comparisons of the area under the curve between daptomycin and daptomycin-loaded cubosomes were determined by 1-way ANOVA followed by Turkey's multiple comparison tests (*P < .05, **P < .01). Error bars show mean ± SD, 3 independent experiments. *B*, Minimum inhibitory concentration of cubosomes in the presence of sub-inhibitory concentrations of daptomycin, vancomycin (VAN), or linezolid (LIN). Negative control (NC) is cubosomes alone.

To compare the bactericidal effects of daptomycin-loaded cubosomes and daptomycin alone, we first selected 14 clinical MRSA strains isolated from patients with bacteremia (Table 1) [8–10, 20–23]. These MRSA isolates represented 2 dominant global MRSA lineages found in hospitals and communities (clonal complex 5 and 8) [8, 36]. Packaging of daptomycin in cubosomes resulted in 2–16 times lower daptomycin MBCs compared with daptomycin alone (Table 1). Time-kill treatment studies mimicking free daptomycin concentrations found in bones and deep tissues using licensed dosing (0.5, 1, and 2 μg/mL) [37, 38], showed that daptomycin and daptomycin-loaded cubosomes were equally bactericidal against all strains within 8 hours (Figure 2*A*). Notably, significantly more bacterial killing was observed at 24 hours for all strains with daptomycin-loaded cubosomes compared with daptomycin alone at various concentrations (Figure 2*A*). Cubosomes alone at equivalent concentrations showed no effects on bacterial growth (Figure 2*A*).

To determine whether the effects of cubosomes were specific to daptomycin, we assessed combinations of cubosomes with 2 other anti-MRSA antimicrobials; vancomycin (cell wall targeting) and linezolid (ribosome inhibition; Figure 2*B*). No additive effect was observed with these antimicrobials, suggesting that the daptomycin-cubosome effect was membrane specific.

### Mechanisms of Interaction Between Daptomycin Packaged Cubosomes and *Staphylococcus aureus*

**Figure 3.**
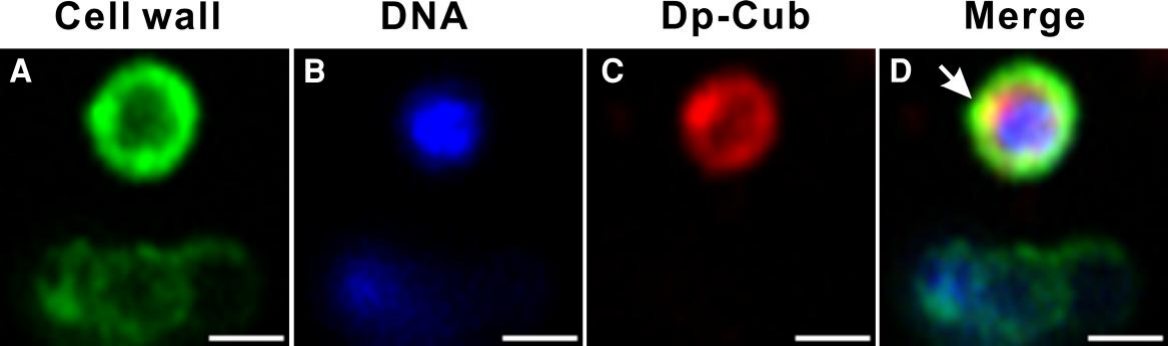
Interactions between *S*. *aureus* A8819 cells and daptomycin-loaded cubosomes. Cells were incubated with daptomycin-loaded cubosomes for 1 h (*A–D*). *S. aureus* cell walls were labeled with wheat germ agglutinin (WGA-488) (*A*). Bacterial DNA was stained with Hoechst (*B*). Daptomycin-loaded cubosomes (Dp-Cub) were labeled with Rhodamine B (*C*). The overlay images between *S*. *aureus* cells and daptomycin-loaded cubosomes are shown in (*D*). The white arrow indicates the diffusion of the cubosomes with the bacterial cell surface. Representative images from 3 biological experiments are shown. The scale bar represents 1 μm.

To characterize the mechanisms of interaction between daptomycin-loaded cubosomes and our clinical MRSA isolates, we first performed super-resolution microscopy using a range of targeted dyes. After 1 hour incubation, daptomycin-loaded cubosomes were observed to associate with the *S. aureus* cell surface (Figure 3*A*–*D*). The daptomycin-loaded cubosomes appeared to diffuse with the sub-cell wall compartment of the bacteria, suggesting integration of daptomycin-loaded cubosomes with the bacterial membrane (Figure 3*D*).

**Figure 4.**
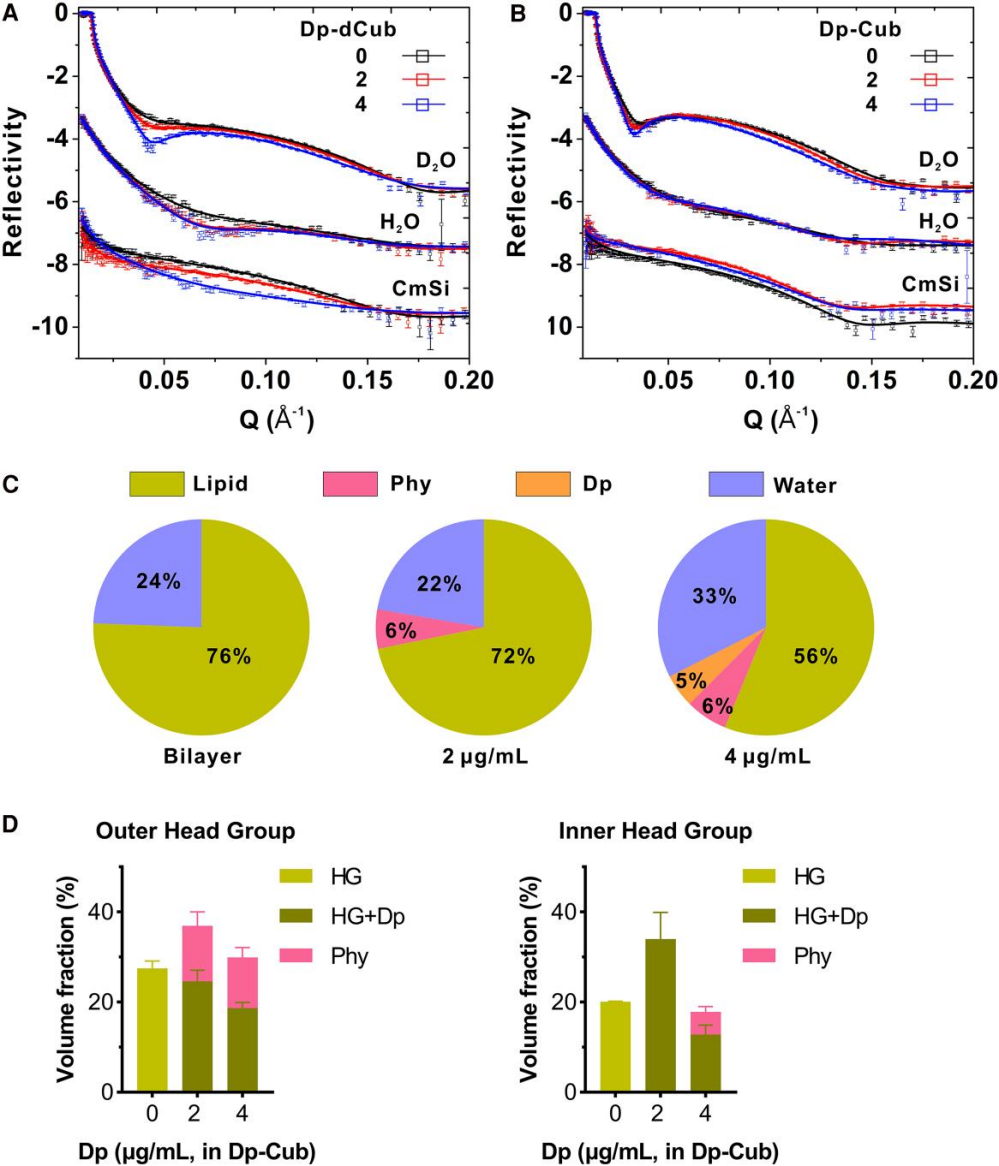
Mechanism of daptomycin-loaded cubosome interaction with *S*. *aureus* membranes. NR profiles (symbols) and fits (lines) of the membranes treated with daptomycin packaged in (*A*) deuterated (to assess for penetration into the tail region) cubosomes (Dp-dCub) or (*B*) protonated (assess for penetration into the head groups) cubosomes (Dp-Cub) in D_2_O, H_2_O, and CmSi are shown. Dosages of 0, 2, and 4 μg/mL daptomycin packaged in cubosomes were used. The NR profiles in H_2_O and CmSi are offset for clarity. *C*, Relative volume fractions of lipid, phytantriol, daptomycin, and water in the tail region of the bilayers before and after incubation with daptomycin packaged in deuterated cubosomes, with calculated errors of ±1%. *D*, Volume fractions of phytantriols, head groups (HGs), and daptomycin at the outer (left) and inner (right) head group regions after treatments of daptomycin-loaded cubosomes are shown. Volume fractions of daptomycin and head groups are shown in sum as the theoretical SLDs between the 2 components were close. Mean ± SD, Monte Carlo analysis.

To confirm these findings, we utilized the high-resolution, membrane characterization approach of NR, which can decipher structural details of bacterial membranes exposed to antimicrobials at ångström (Å) level in biologically relevant buffer systems [26, 27]. To enable precise quantitation of cubosomes penetrating into the *S. aureus* membrane, we encapsulated daptomycin within either deuterated (for penetration into the tail region) or protonated (for penetration into the head groups) cubosomes (Supplementary Figure 3*A* and 3*B*) [24]. We reconstituted the membrane bilayer of a clinical MRSA strain (A8819) that caused a complicated human infection with bacteremia and osteomyelitis using the previously reported phospholipid composition [26], and quantified the interactions between membranes and daptomycin-loaded cubosomes using NR (Figure 4*A* and 4*B*). At low concentrations of daptomycin packaged within cubosomes (2 μg/mL), no direct penetration of daptomycin into the *S. aureus* membrane was observed, but a significant reduction in lipid volume was seen, suggesting the initiation of a lipid extraction mechanism [26] (Figure 4*C*, Supplementary Tables 2 and 3, Supplementary Figure 4). Notably, the cubosome lipids (phytantriols) accumulated exclusively on the outer leaflet of the bacterial membranes (Figure 4*D*, Supplementary Table 3), indicating a selective interaction without deeper integration at this concentration. The effects became significantly more pronounced when daptomycin concentrations were increased to 4 μg/mL within cubosomes (Figure 4*C*, Supplementary Table 3). Under these conditions, daptomycin was not only able to penetrate the membrane but also actively dislocate acyl-chains, indicating a direct interaction with the lipid bilayer structure. In addition, the cubosome lipids (phytantriol) transitioned from their previous restriction to the outer leaflet, to now being able to penetrate deeper into the membrane bilayer (Figure 4*C*, Supplementary Table 3). This deeper integration allowed phytantriols to reach the inner leaflet or even the head group regions of the membrane (Figure 4*D*, Supplementary Table 3, Supplementary Figure 4), highlighting a cooperative mechanism between daptomycin and cubosomes in destabilizing the bacterial membrane architecture. Taken together, these data showed that daptomycin-loaded cubosomes led to membrane targeting and penetration of daptomycin, with associated lipid extraction and membrane damage, as well as cubosome lipid integration across the whole bacterial membrane bilayer.

### Daptomycin Pretreatment Facilitated the Insertion of Cubosome Lipids into *Staphylococcus aureus* Membranes

**Figure 5.**
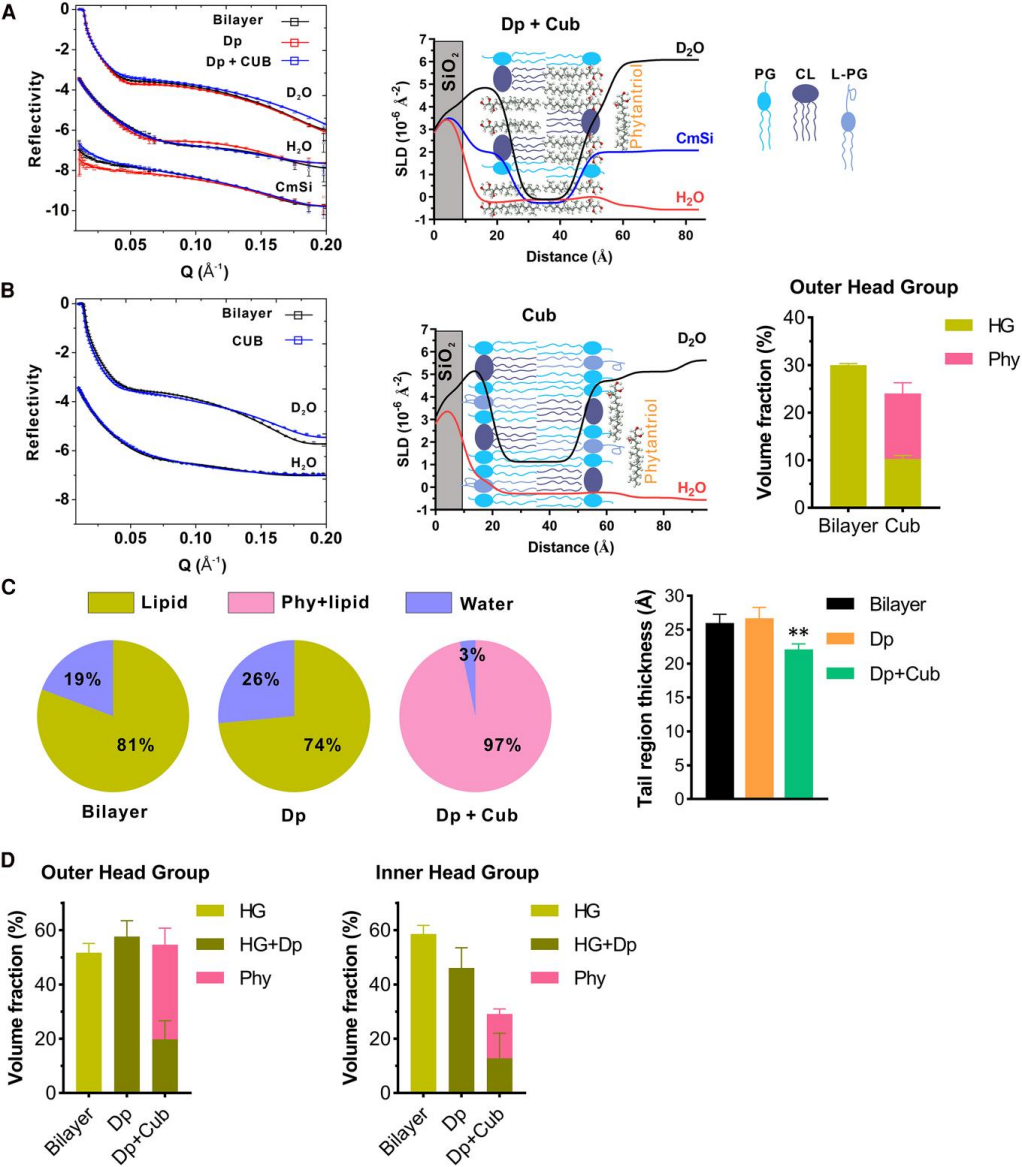
Daptomycin-dependent cubosome actions on *S*. *aureus* membranes. *A*, NR profiles (symbols) and fits (lines) of membranes treated with 2 μg/mL daptomycin followed by 32 μg/mL cubosomes in D_2_O, H_2_O, or CmSi are shown (left). The NR profiles in H_2_O and CmSi are offset for clarity. The corresponding scattering length densities (SLD) profiles and schematic diagram of the membrane are shown (right). CL, cardiolipin. PG, phosphatidylglycerol. L-PG, lysyl-phosphatidylglycerol. *B*, NR profiles (left) and the SLD profiles (middle) of membranes treated with 32 μg/mL cubosomes alone are shown. Volume fractions of phytantriols, head groups and tails at the outer head group are shown (right). *C*, Relative volume fractions of lipid, phytantriol, and water in the tail region of the bilayers before and after incubation with daptomycin followed by cubosomes, with calculated errors of ±1%. Volume fractions of phytantriols and membrane tails are shown in sum as the theoretical SLDs between the 2 components were close. Thickness of the tail regions is shown (right). **P < .01, Welch's *t*-test compared with the Bilayer. *D*, Volume fractions of phytantriols, head groups and daptomycin at the outer (left) and inner (right) head group regions after the incubation with 2 μg/mL daptomycin followed by 32 μg/mL cubosomes. Volume fractions of daptomycin and head groups are shown in sum as the theoretical SLDs between the 2 components are close. Mean ± SD, Monte Carlo analysis.

To investigate if phytantriol insertion into bacterial membranes was daptomycin dependent, we treated our reconstituted MRSA membranes with daptomycin followed by cubosomes (Figure 5*A*, Supplementary Figure 5*A*) or cubosomes alone (Figure 5*B*, Supplementary Figure 5*B*). First, membranes were treated with daptomycin (2 μg/mL), which led to membrane lipid extraction (Figure 5*C*), as reported previously [26]. We then exposed these daptomycin-affected membranes to cubosomes (32 μg/mL), and showed that phytantriols accumulated within the bilayer tail region, leading to a significant increase in the lipid content of the membrane (Figure 5*A* and 5*C*), with structural disruption as evidenced by a decrease in the thickness of the tail region due to the short tails of phytantriols (Figure 5*C*). Phytantriols were also seen at the outer and inner head group regions (Figure 5*D*), confirming that the cubosome lipids penetrated and accumulated across the entire membrane bilayer in the context of daptomycin-affected membranes. Notably, treatment of the MRSA membranes with cubosomes alone (32 μg/mL) led to the cubosomes only associating with the outer head groups of the membrane (Figure 5*B*), with no penetration into the tail or inner head group region (Figure 5*B*, Supplementary Figure 5*C*) and no thickness reduction of the membrane tail region (Supplementary Figure 5*C*). Together, these data confirmed that the cubosome lipids were able to infuse and penetrate staphylococcal membranes only in the setting of daptomycin-membrane interactions, providing mechanistic insights into the synergy between daptomycin and cubosomes as observed in vitro (Table 1, Figure 2).

### In Vivo Efficacy of Daptomycin Packaged Cubosomes

**Figure 6.**
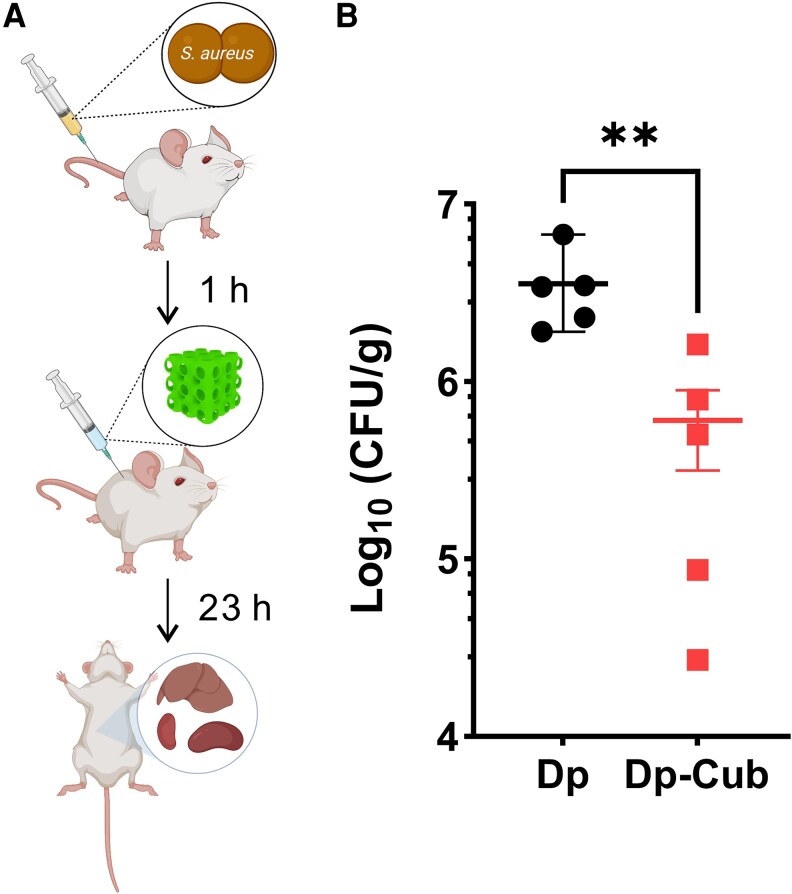
In vivo efficacy of daptomycin-loaded cubosomes and daptomycin in a murine bacteremia model. *A*, Schematic of the experimental protocol for the murine bacteremia model. Created in BioRender. Jiang, J. (2025) https://BioRender.com/9xca2yk. *B*, Colony forming units (c.f.u.) of *S*. *aureus* (A8819) found in organs of infected mice 23 h after treatment with daptomycin (Dp, 10 mg/kg) or 18.0 wt% daptomycin-loaded cubosomes (Dp-Cub, daptomycin 10 mg/kg). Five mice per group; Error bars show mean ± SEM. Daptomycin-loaded cubosomes was compared with daptomycin by Mann–Whitney test (**P < .01).

To further confirm the in vitro findings, the effectiveness of daptomycin-loaded cubosomes to treat MRSA infections was evaluated in a murine staphylococcal bacteremia model [39] (Figure 6*A*). Daptomycin is licensed as a daily dose of 6 mg/kg to treat MRSA infection in humans [10], which is equivalent to 50 mg/kg when used in mice [40]. To mimic sub-optimal therapeutic daptomycin concentrations in vivo [41], mice with bacteremia were treated at 1 hour postinfection with 10 mg/kg daptomycin. This was compared with equivalent daptomycin concentrations packaged within cubosomes (18.0 wt% daptomycin-loaded cubosomes), equivalent concentrations of unloaded cubosomes (45.6 mg/kg) and a saline control. As shown in Figure 6*B*, daptomycin-loaded cubosomes significantly reduced the bacterial burden in the organs of infected mice compared with daptomycin alone (*P* = .0079). All infected mice treated with cubosomes alone or saline showed signs of animal distress and were euthanized before 16 hours (data not shown). Taken together, these data support further investigation of daptomycin packaged within cubosomes as a future therapeutic strategy to treat MRSA infections.

## DISCUSSION

In the recent global assessment of the burden of antimicrobial resistance, MRSA was shown to be the pathogen with the highest associated mortality [42]. New solutions and innovative therapeutic strategies are urgently required. Here, we report on cubic, lipid nanoparticles known as cubosomes as carriers of the last line anti-staphylococcal antibiotic, daptomycin, to treat severe infections caused by multidrug-resistant *S*. *aureus*. We showed that daptomycin could be encapsulated within cubosomes at a high loading capacity, leading to sustained drug release with preserved internal cubic-phase structure of the cubosome under physiologically relevant conditions. Daptomycin packaged within cubosomes demonstrated therapeutic advantages in killing clinical MRSA isolates compared with daptomycin or cubosomes alone, and this was confirmed in vitro and within an MRSA bacteremia model. The synergy between daptomycin and cubosomes against *S*. *aureus* was initiated by the docking of cubosomes on bacterial cells, followed by daptomycin release and targeting of the bacterial membranes, resulting in lipid extraction and membrane solvation. Finally, daptomycin-affected bacterial membranes then potentiated the integration of the cubosome lipids throughout the *S. aureus* bilayer membrane, confirming the specificity of this unique lipid targeting nanocarrier with daptomycin against staphylococcal infections.

The synergistic antibacterial activity with cubosomes was observed exclusively in combination with daptomycin, but not with vancomycin or linezolid, indicating that the membrane targeting properties of daptomycin were responsible for this effect. Interestingly, despite the membrane targeting of the cationic antimicrobial peptide, LL37, and the polymyxin antibiotics, packaging of these agents in cubosomes reduced their bactericidal effects [43, 44], suggesting that the synergy between daptomycin and cubosomes is unique to this partnership. Advanced techniques are now available to decipher the detailed mechanisms of drug-membrane interactions, more specifically, membrane models with biologically relevant lipid compositions and neutron scattering techniques that allow for direct structural analyses [26]. Using NR to analyze reconstituted MRSA membranes, we found that daptomycin-packaged cubosomes led to membrane acyl-chain dislocation, solubilization, and cubosome lipid (phytantriol) integration, explaining our microbiological findings of the synergy between daptomycin and cubosomes. We showed that cubosomes alone did not integrate into bacterial membranes but instead accumulated on the membrane surface. Cubosome lipid (phytantriol)-membrane integration was only observed in daptomycin-affected membranes, indicating that the synergy between daptomycin and cubosomes is initiated by daptomycin-mediated lipid membrane disruption, enabling subsequent phytantriol membrane integration. Disrupted membrane stability caused by phytantriol integration may further enhance the multifaceted bactericidal effects of daptomycin, including membrane depolarization and inhibition of cell wall biosynthesis [6]. Dyett et al. showed that encapsulation of antibiotics in cubosomes improves drug delivery and enhanced antibacterial effects for novobiocin and piperacillin, but not for meropenem or gentamicin [45], suggesting that the advantages for cubosome encapsulation may depend on the mechanism of action of the antibiotic.

There is increasing interest in packaging and augmenting the antimicrobial activity of daptomycin. Previous studies have attempted to load daptomycin into chitosan nanoparticles and liposomes but have had challenges, with reduced antimicrobial activity, rapid drug release, and a low capacity for daptomycin loading [46–48]. Other studies have reported that conjugation of daptomycin with silver or gold nanoclusters enhanced bactericidal effects of daptomycin in vitro [49], but the intrinsic toxicity of silver ions raises concerns for in vivo antimicrobial efficacy. In the current study, daptomycin encapsulated within phytantriol-based cubosomes demonstrated highly stable and sustainable daptomycin release kinetics over 72 hours, and showed enhanced antibacterial effects in vitro and in a bacteremia model, confirming that cubosomes acted as a bifunctional daptomycin carrier and potentiator in vivo. This current study is the first step in evaluating this nanocarrier formulation, and more studies are needed, including testing the effects of daptomycin-packaged cubosomes on other *S*. *aureus* lineages. Given that the median duration of successful daptomycin treatment for MRSA infective endocarditis is 27 days [50], further studies are also needed to explore the pharmacokinetics of daptomycin-packaged cubosomes over longer periods of time. Furthermore, the impact on toxicity, particularly myotoxicity, of daptomycin packaged within a lipid nanostructure is needed, as the role of cubosomes and muscle cell interactions remains unknown. Based on the specific engineering of our cubosomes with a high bacterial membrane affinity lipid (phytantriol), it is plausible that cubosomes may allow for higher daptomycin concentrations with less toxicity, however this needs further validation.

In summary, we showed that daptomycin packaged in phytantriol-based cubosomes was synergistic in killing MRSA in vitro and in vivo, and this was via a specific and combined bacterial membrane targeting mechanism. Our study highlights a potential innovative therapeutic strategy to utilize a smart, lipid nanocarrier for delivering a critical antibiotic, daptomycin, directly to its target site to have impact on a globally important, multidrug-resistant bacterial pathogen.

## Supplementary Material

jiaf492_Supplementary_Data

## Supplementary Data

Supplementary materials are available at The Journal of Infectious Diseases online (http://jid.oxfordjournals.org/). Supplementary materials consist of data provided by the author that are published to benefit the reader. The posted materials are not copyedited. The contents of all supplementary data are the sole responsibility of the authors. Questions or messages regarding errors should be addressed to the author.
