# Supplementary material for: Daptomycin-Loaded Nanocarriers Facilitate Synergistic Killing of Methicillin-Resistant Staphylococcus aureus via Lipid-Mediated Interactions and Targeting
Source: J Infect Dis. 2025 Nov 4;233(1):e22–33. doi: 10.1093/infdis/jiaf492 (PMC12811857; doi:10.1093/infdis/jiaf492)
Supplement: jiaf492_Supplementary_Data [file jiaf492_supplementary_data.docx]

**Supporting Information**

**Supplementary Methods**

### **Small-angle X-ray scattering (SAXS)**

SAXS measurement were carried out at the SAXS/wide-angle X-ray scattering (WAXS) beamline at the Australian Synchrotron (Clayton, Victoria), with 1.24 Å wavelength and beam flux of approximate 10^13^ photons per second. Silver behenate (d-spacing = 58.38 Å) was used as a standard. Cubosomes were loaded in 1.0 mm quartz capillaries (Hampton Research, United States), followed by high throughput scanning of X-ray beamline with one second of exposure time. 2D scattering patterns of cubosomes were captured using a Dectris-Pilatus-1M CCD camera. Data analysis of the 2D scattering images was carried out using a custom-written IDL-based AXcess software [1].

### **Cryo-transmission electron microscopy (Cryo-TEM)**

To prepare hydrophilic carbon-supported copper grids for the Cryo-TEM, copper grids (200-mesh) were coated with perforated carbon film (Lacey carbon film, ProSciTech, Qld, Australia) and glow discharged in nitrogen. A droplet of cubosomes was placed onto each grid, followed by gentle blotting of the grid by filter paper. The grid was then rapidly propelled into liquid ethane at its freezing point for the specimen vitrification. The samples were imaged using a Tecnai 12 Cryo-Transmission Electron Microscope (TEM) (FEI, Eindhoven, Netherlands) with a Gatan 626 cryo holder (Gatan Pleasanton, CA, USA) at 120 kV and 8-10 electrons/Å^2^. Images were collected with a Megaview III CCD camera using AnalySIS software (Olympus) with magnifications ranging from 30000X to 97000X.

### **Super-resolution microscopy**

Fresh *S. aureus* overnight cultures were diluted to a final inoculum of 10^9^ CFU/mL in CaMHB. The bacterial cell surface was stained with 5 μL of 2 mg/mL Wheat Germ Agglutinin-Alexa Fluor™ 488 and incubated for 10 minutes at 37 °C with constant shaking at 200 rpm. The cells were collected by centrifugation and washed with CaMHB. The bacterial suspension was then incubated with RhD-B-labelled daptomycin-loaded cubosomes. After the incubation, 10 µL of bacterial suspension was transferred to the poly-L-lysine coated coverslip (Carl Zeiss Microscopy GmbH, Jena, Germany). Live cell imaging of *S. aureus* cells was acquired using a Zeiss LSM 980 Airyscan 2 microscope (Carl Zeiss Microscopy GmbH, Jena, Germany) equipped with a 32+2-Channel GaAsP detector and an Airyscan 2 detector. Samples were imaged in Airyscan 2 super-resolution mode using a 63× oil C PlanApo 1.4NA objective with 488 nm laser power of 4 % and 561 nm laser power of 4 %. The images were processed in ImageJ.

### **Neutron reflectivity of *S. aureus* bilayer membrane**

*S. aureus* membrane bilayers were formed on silicon oxide (SiO_2_) surfaces using published methods [2]. Lipids—1,2-dioleoyl-sn-glycero-3-phospho-(1'-rac-glycerol), 1',3'-bis[1,2-dioleoyl-sn-glycero-3-phospho]-glycerol, and 1,2-dioleoyl-sn-glycero-3-[phospho-rac-(3-lysyl(1-glycerol))]—were mixed at a 69:12:19 molar ratio. A custom Langmuir-Blodgett trough was used to assemble the bilayers [3]. Specular neutron reflectivity was measured after 4 h daptomycin-packaged cubosomes treatment using the Platypus and Spatz time-of-flight reflectometer (2.8-18 Å spectrum range) at the OPAL reactor (Australia) [2, 4, 5]. Data were collected on a 2D ^3^He detector with (60 mm footprint) at 0.85° and 3.5° incident angles in 100 % D_2_O, 100 % H_2_O, and contrast match silicon (CmSi, 62% H_2_O/38% D_2_O). Data were reduced using refnx [6], and reflectivity was plotted as a function of momentum transfer, *Q* = *4π sin θ / λ*.

NR analysis was carried out using the Refnx MOTOFIT [7]. The bilayer was modeled as three slabs: inner leaflet headgroups, tails, and outer leaflet headgroups. Theoretical neutron scattering length densities (SLDs, 10^-6^ Å^-2^) in D_2_O, H_2_O, and CmSi were: solvent (6.35, -0.56, 2.07), head groups (3.57, 2.65, 3.00), tails (-0.28, -0.28, -0.28), daptomycin (3.96, 2.41, 3.00), deuterated phytantriol (7.63, 6.83, 7.03), protonated phytantriol (0.365, -0.388, -0.102), and Si (2.07 for all). Slabs under different isotopic contrasts (D_2_O, H_2_O) were fitted simultaneously to minimize χ2 using a differential evolution algorithm. The best-fit model provided SLD, thickness and roughness of each slab. For slabs with two components (species *s* and water *w*), SLD was calculated as:

*P_layer_ = ∅ P_s_ + (1−∅) P_w_*

where *∅* is the volume fraction of species s. *P_w_* and *P_s_* are the SLDs of the two components. Parameters uncertainties were estimated using a Bayesian Markov Chain Monte Carlo (MCMC) approach on the best-fit models. The MCMC used 200 walkers over 50 steps with a thinning factor of 400, generating 20,000 fits [8]. Frequency plot of fitted values were analyzed, and errors were defined as twice the standard deviation, with the parameter values taken as the midpoint of the 95% confidence interval.

**Table S1.** Composition of cubosomes, the particle hydrodynamic diameter, and lattice parameter derived from DLS and SAXS measurements

| Dp^a^ loading (wt%) | 0 | 4.5 | 9.0 | 13.5 | 18.0 | 22.5 |
| --- | --- | --- | --- | --- | --- | --- |
| Average diameter (nm) | 168.8 ± 10.6 | 225.5 ± 21.7 | 197.6 ± 19.8 | 181.8 ± 13.2 | 152.4 ± 5.8 | 179.5 ± 10.4 |
| PDI^b^ | 0.25 ± 0.07 | 0.16 ± 0.01 | 0.18 ± 0.01 | 0.08 ± 0.02 | 0.11 ± 0.02 | 0.07 ± 0.01 |
| Zeta potential (mV) | -24.9 ± 0.5 | -27.4 ± 0.8 | -26.0 ± 1.4 | -30.6 ± 2.9 | -36.5 ± 0.4 | -28.6 ± 0.9 |
| Lattice parameter^c^ (Å) | 65.3 | 73.6 | 78.7/101.4 | 109.7 | 125.7 | − |
| Internal structure | Cubic (Pn3m) | Cubic (Pn3m) | Cubic (Pn3m/Im3m) | Cubic (Im3m) | Cubic (Im3m) | Sponge (Disorder cubic phase) |

1. Dp, daptomycin
2. PDI, polydispersity index
3. The repeated distance of the membrane and the water channel

Table S2. Substrate and bilayer properties of reconstituted A8819 membranes treated with daptomycin packaged in deuterated cubosomes (Dp-dCub). Error is one standard deviation.

| Layer | Thickness  (Å) | nSLD^a^  (🞨10^-6^ Å^-2^) | | | V_f_^b^ (%) | | | R^c^  (Å) |
| --- | --- | --- | --- | --- | --- | --- | --- | --- |
|  |  | D_2_O^d^ | H_2_O^d^ | CmSi^d^ | Lipid | Dp^e^ | Phy^f^ | - |
| **Before treatment** | | | | | | | | |
| SiO_2_ | 13.1 ± 1.1 | 3.81 ± 0.01 | 2.87 ± 0.08 | 3.43 ± 0.12 | - | - | - | 3 |
| IHG^g^ | 11.6 ± 1.2 | 4.77 ± 0.13 | 1.10 ± 0.17 | 2.55 ± 0.05 | 51.7 ± 5.5 | - | - | 3 |
| Tails | 31.7 ± 0.8 | 1.26 ± 0.02 | -0.35 ± 0.00 | 0.29 ± 0.01 | 75.6 ± 0.3 | - | - | 3 |
| OHG^g^ | 6.6 ± 0.5 | 5.27 ± 0.04 | 0.45 ± 0.05 | 2.36 ± 0.01 | 31.6 ± 1.6 | - | - | 3 |
| **Dp-dCub** **at 2 μg/mL daptomycin** | | | | | | | | |
| SiO_2_ | 13.1 ± 1.1 | 3.81 ± 0.01 | 2.87 ± 0.08 | 3.43 ± 0.12 | - | - |  | 3 |
| IHG | 15.3 ± 1.6 | 4.64 ± 0.23 | 1.25 ± 0.33 | 2.29 ± 0.18 | 56.4 ± 10.3^h^ | | - | 3 |
| Tails | 27.3 ± 2.0 | 1.60 ± 0.07 | 0.08 ± 0.08 | 0.79 ± 0.18 | 71.8 ± 1.1** | - | 5.9 ± 1.1 | 3 |
| OHG | 9.2 ± 1.6 | 4.48 ± 0.47 | 0.77 ± 0.33 | 2.11 ± 0.05 | 41.4 ± 10.3 | | - | 3 |
| **Dp-dCub at 4 μg/mL daptomycin** | | | | | | | | |
| SiO_2_ | 13.1 ± 1.1 | 3.81 ± 0.01 | 2.87 ± 0.08 | 3.43 ± 0.12 | - | - | - | 3 |
| IHG | 17.6 ± 1.8 | 4.94 ± 0.21 | 0.57 ± 0.22 | 2.93 ± 0.20 | 36.8 ± 5.3 | | - | 3 |
| Tails | 27.9 ± 3.2 | 2.53 ± 0.08 | 0.21 ± 0.05 | 2.25 ± 0.04 | 57.0 ± 1.2** | 5.0 ± 0.7 | 6.2 ± 0.7 | 3 |
| OHG | 7.9 ± 1.9 | 5.10 ± 1.09 | 0.18 ± 0.38 | 2.54 ± 0.16 | 28.8 ± 6.1 | | - | 3 |
| Phy | 16.4 ± 3.2 | 5.96 ± 0.14 | -0.52 ± 0.04 | 2.24 ± 0.06 | - | - | 0.5 ± 0.5 | 3 |

a. nSLD, neutron scattering length density, the fitted D_2_O SLD is 6.05 × 10^-6^ Å^-2^.

b. V_f_, volume fraction.

c. R, roughness

d. Solvent used in the measurement

e. Dp, daptomycin

f. Phy, phytantriol

g. HGs, head groups of the inner leaflet (IHG) and the outer leaflet (OHG).

h. Calculation of lipid volume fraction in IHG and OHG was not achievable as the theoretical SLDs of daptomycin and HGs are close.

***P* < 0.01, Welch’s *t*-test compared with untreated membrane.

Table S3. Substrate and bilayer properties of reconstituted A8819 membranes treated with daptomycin-loaded cubosomes (Dp-Cub). Error is one standard deviation.

| Layer | Thickness  (Å) | nSLD^a^  (🞨10^-6^ Å^-2^) | | | V_f_^b^ (%) | | | | R^c^  (Å) |
| --- | --- | --- | --- | --- | --- | --- | --- | --- | --- |
|  |  | D_2_O^d^ | H_2_O^d^ | CmSi^d^ | Lipid | Phy^e^ | Dp^f^ | | - |
| **Before treatment** | | | | | | | | | |
| SiO_2_ | 19.5 ± 0.2 | 4.2 ± 0.00 | 2.87 ± 0.08 | 3.43 ± 0.12 | - | - | | - | 3 |
| IHG^g^ | 16 ± 0.0 | 5.58 ± 0.00 | 0.09 ± 0.00 | 2.19 ± 0.00 | 20.1 ± 0.1 | - | | - | 3 |
| Tails | 33.2 ± 0.2 | 1.59 ± 0.01 | -0.36 ± 0.00 | 0.38 ± 0.00 | 70.6 ± 0.2 | - | | - | 3 |
| OHG^g^ | 6.1 ± 0.2 | 5.40 ± 0.04 | 0.32 ± 0.05 | 2.26 ± 0.02 | 27.5 ± 1.6 | - | | - | 3 |
| **Dp-Cub at 2 μg/mL daptomycin** | | | | | | | | | |
| SiO_2_ | 19.5 ± 0.2 | 4.2 ± 0.00 | 2.87 ± 0.08 | 3.43 ± 0.12 | - | - | | - | 3 |
| IHG | 18.5 ± 0.6 | 5.76 ± 0.09 | 0.53 ± 0.19 | 2.19 ± 0.14 | 34.0 ± 5.9 (+Dp)^h^ | - | | - | 3 |
| Tails | 31.3 ± 0.7 | 1.74 ± 0.04 | -0.41 ± 0.04 | 0.42 ± 0.13 | 68.0 ± 0.6^i^ | | | - | 3 |
| OHG | 14.1 ± 1.0 | 4.73 ± 0.17 | 0.25 ± 0.08 | 2.23 ± 0.12 | 24.6 ± 2.5 | 12.3 ± 3.1 | | - | 3 |
| **Dp-Cub at 4 μg/mL daptomycin** | | | | | | | | | |
| SiO_2_ | 19.5 ± 0.2 | 4.2 ± 0.00 | 2.87 ± 0.08 | 3.43 ± 0.12 | - | - | | | 3 |
| IHG | 19.8 ± 0.3 | 5.64 ± 0.05 | -0.13 ± 0.06 | 2.90 ± 0.10 | 13.1 ± 2.1  (+Dp)^h^ | 5.2 ± 0.9 | | - | 3 |
| Tails | 33.2 ± 0.5 | 2.38 ± 0.02 | -0.17 ± 0.03 | 0.25 ± 0.22 | 56.9 ± 2.5^i^ | | | 7.8 ± 1.0 | 3 |
| OHG | 18.1 ± 0.9 | 5.20 ± 0.10 | 0.06 ± 0.04 | 1.91 ± 0.08 | 18.8 ± 1.2  (+Dp)^h^ | 10.0 ± 1.7 | | - | 3 |
| Phy | 19.8 ± 0.2 | 5.87 ± 0.04 | -0.51 ± 0.03 | 1.88 ± 0.07 | - | 7.4 ± 0.7 | | - | 3 |

a. nSLD, neutron scattering length density, the fitted D_2_O SLD is 6.09 × 10^-6^ Å^-2^.

b. V_f_, volume fraction.

c. R, roughness

d. Solvent used in the measurement

e. Phy, Phytantriol

f. Dp, daptomycin

g. HGs, head groups of the inner leaflet (IHG) and the outer leaflet (OHG).

h. Calculation of lipid volume fraction in the HGs was not achievable as the theoretical SLDs of daptomycin and HGs are close.

i. Calculation of lipid volume fraction in tail region was not achievable as the theoretical SLDs of tails and protonated phytantriol are close.


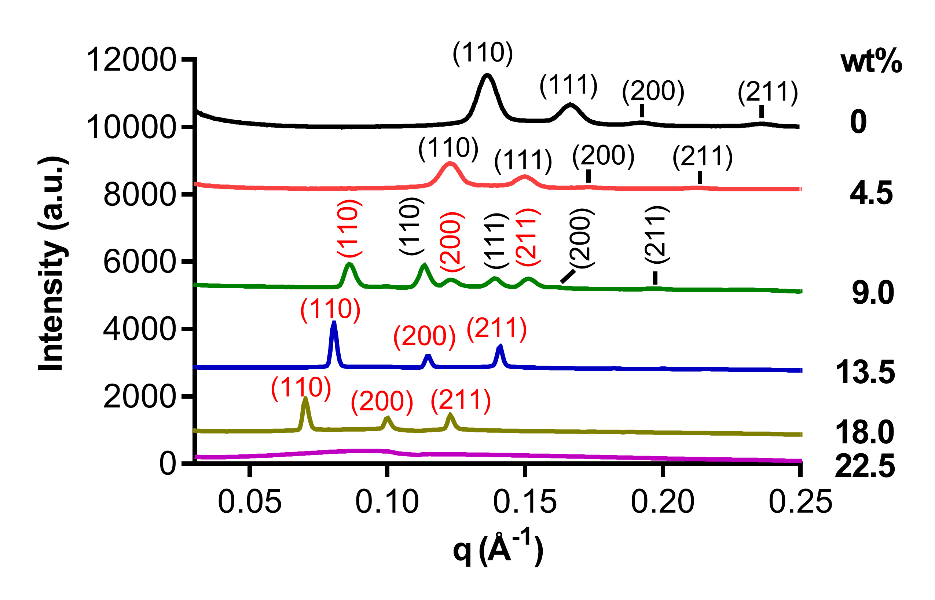


**Figure S1.** SAXS profile of unloaded cubosomes or daptomycin-loaded cubosomes (mass fraction 4.5, 9.0, 13.5, 18.0 and 22.5 wt%) in saline. Bragg reflections representing Pn3m (diamond) or Im3m (primitive) cubic phase structures are indicated with Miller’s indices shown in black and red, respectively. The Intensity (Y-axis) is offset for clarity. No Pn3m or Im3m Bragg reflections were found in 22.5 wt% daptomycin-loaded cubosomes.


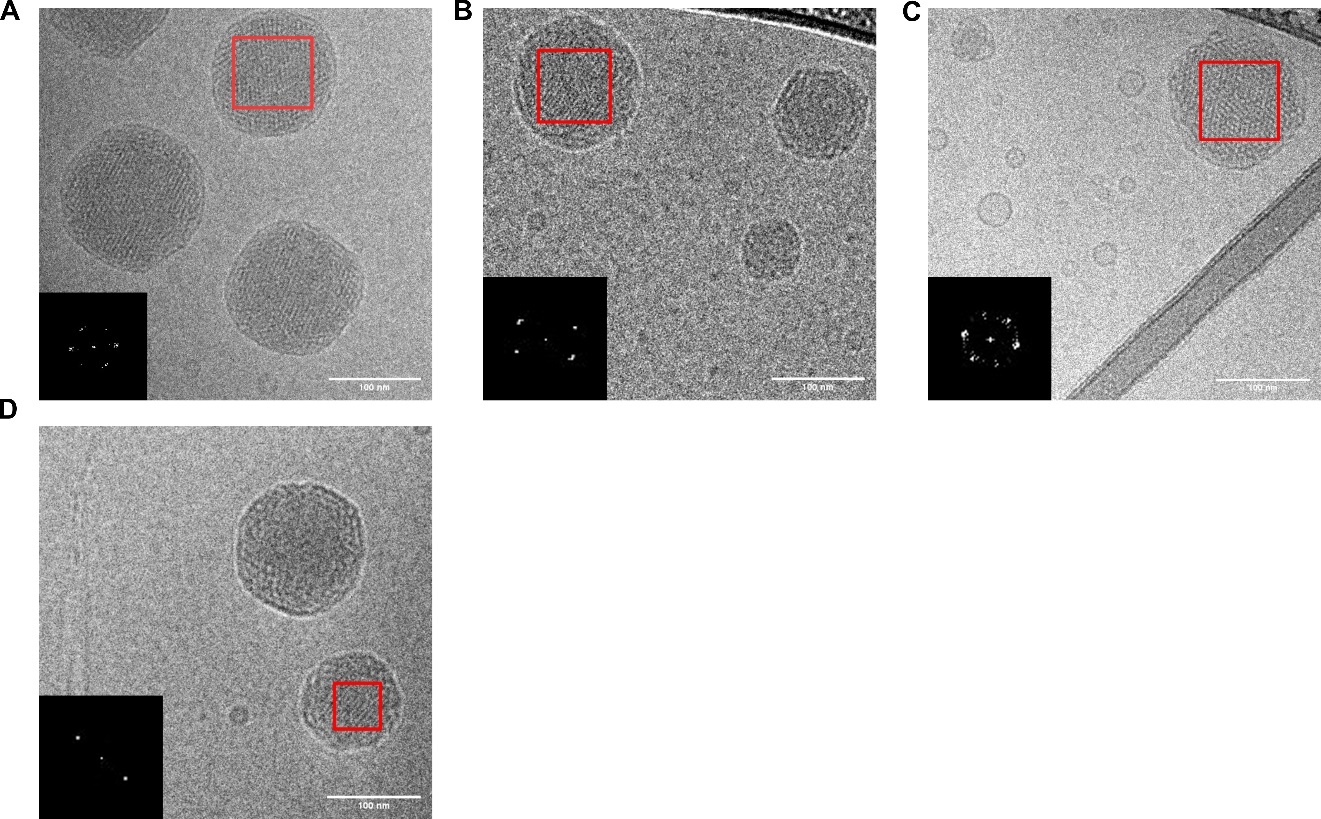


**Figure S2.** Representative cryo-TEM images of cubosomes loaded with daptomycin at (**A**) 0 wt% (**B**) 4.5 wt%, (**C**) 9 wt% and **(D)** 13.5 wt% are shown. The insets display their Fast Fourier Transform analyses of the dispersed particles (red rectangles) of internal structural symmetry. The scale bar is 100 nm.


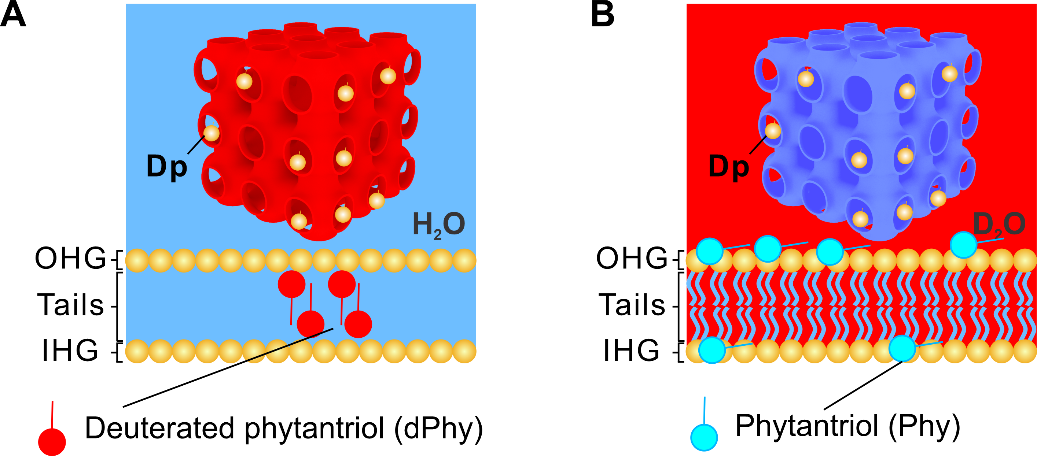


**Figure S3.** Measurement of interactions between cubosomes and the *S*. *aureus* membrane using neutron reflectometry (NR) (**A**) In H_2_O buffer, deuterated phytantriol from daptomycin packaged in deuterated cubosomes can be observed within the membrane tails region, allowing precise quantification of cubosomes penetration into the membrane. (**B**) In D_2_O buffer, protonated phytantriols at outer and inner head groups (OHG and IHG) of the membrane can be measured, providing quantification of cubosomes infusion across the membrane.


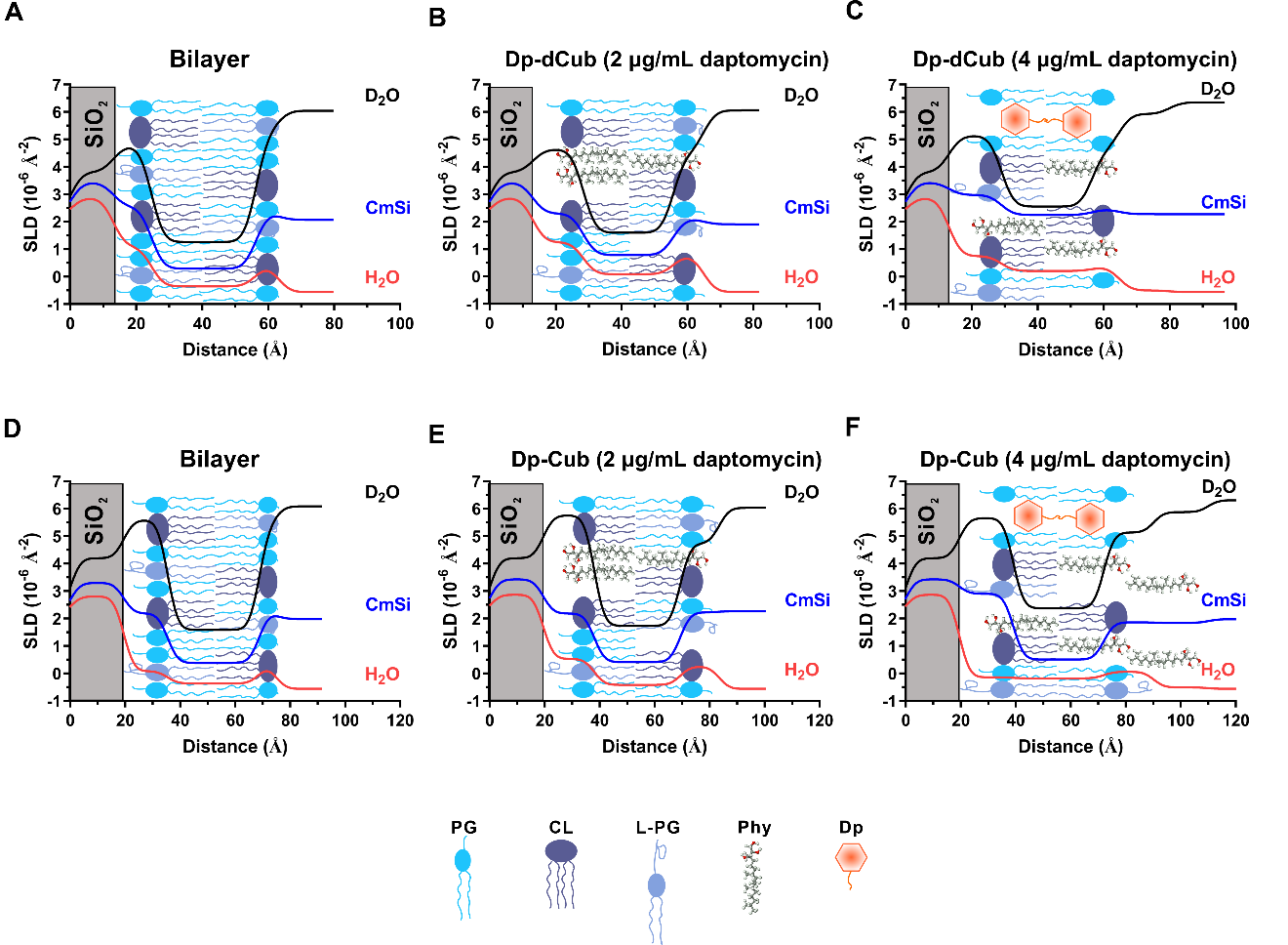


**Figure S4.** The corresponding scattering length densities (SLD) profiles of biomimetic *S*. *aureus* membranes treated with daptomycin packaged in deuterated or protonated cubosomes. (**A**) Bilayer membranes before treatment and after treated with (**B**) 2 μg/mL or (**C**) 4 μg/mL daptomycin packaged in deuterated cubosomes (Dp-dCub). (**D**) Bilayer membranes before treatment and after treated with (**E**) 2 μg/mL or (**F**) 4 μg/mL daptomycin packaged in cubosomes (Dp-Cub). In SLD profiles, the lipids with four tails represent TOCL. The lipids with small head groups represent DOPG whilst the lipids with large head group represent Lys-DOPG. Phytantriol (Phy) and daptomycin (Dp) are shown.

**
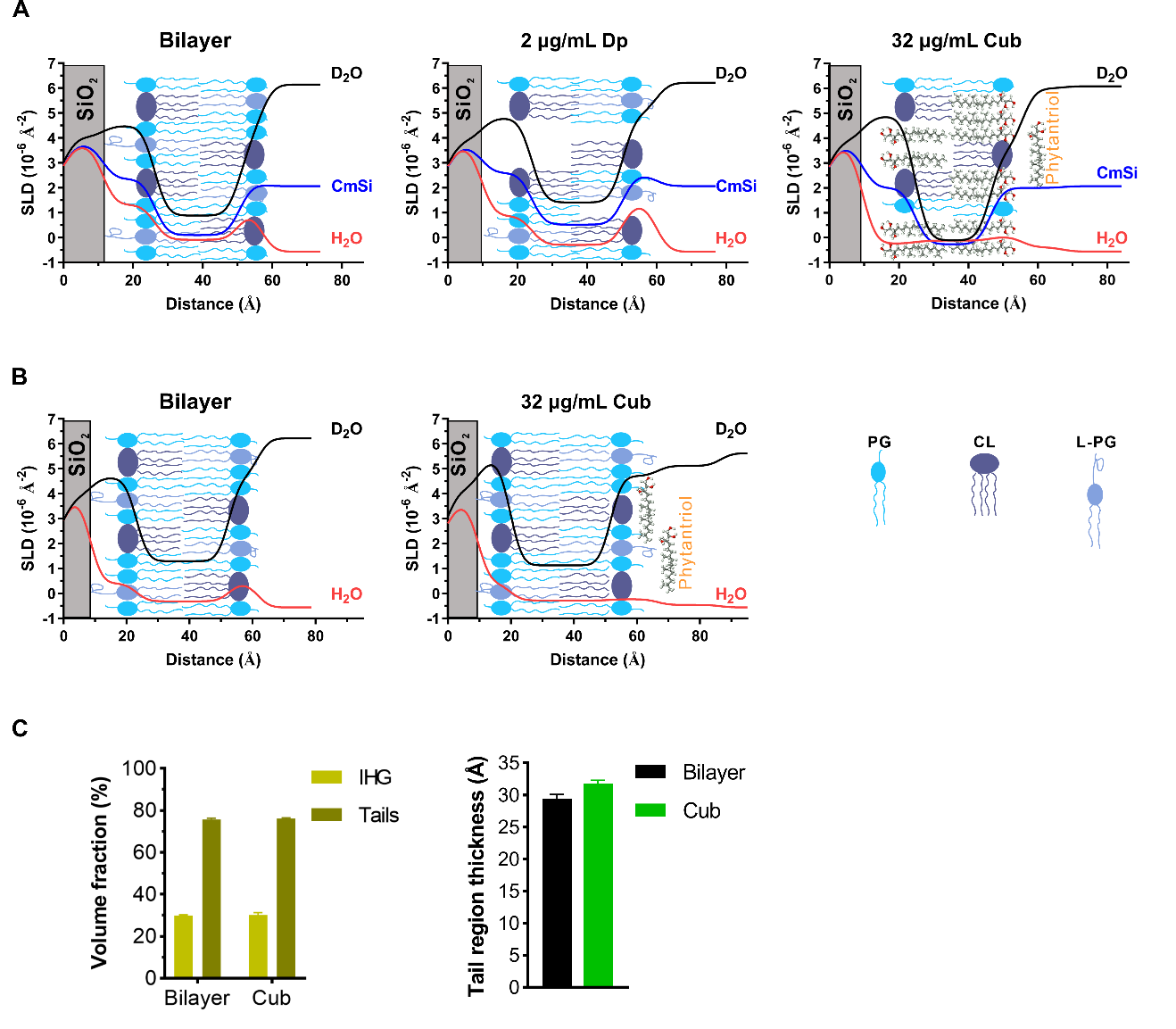
**

**Figure S5.** Interactions between *S*. *aureus* cells and cubosomes. (**A**) The corresponding scattering length densities (SLD) profiles of biomimetic *S*. *aureus* membranes before (left) and after the treatment with daptomycin at 2 μg/mL (middle), followed by cubosomes at 32 μg/mL (right). (**B**) The corresponding SLD profiles of biomimetic *S*. *aureus* membranes before (left) and after the treatment with cubosomes at 32 μg/mL (right). In SLD profiles, the lipids with four tails represent cardiolipin (CL). The lipids with small head groups represent phosphatidylglycerol (PG) whilst the lipids with large head group represent lysyl-phosphatidylglycerol (L-PG). (**C**) For the treatment of 32 μg/mL cubosomes alone, volume fractions of phytantriols, head groups and tails at the inner head group (IHG) and the tail regions are shown (left). Thickness of the tail regions before and after the treatment of 32 μg/mL cubosomes is shown (right).

References

1. Seddon JM, Squires AM, Conn CE, et al. Pressure-jump X-ray studies of liquid crystal transitions in lipids. Philosophical Transactions of the Royal Society A: Mathematical, Physical and Engineering Sciences **2006**; 364:2635–55.

2. Jiang JH, Bhuiyan MS, Shen HH, et al. Antibiotic resistance and host immune evasion in *Staphylococcus aureus* mediated by a metabolic adaptation. Proceedings of the National Academy of Sciences of the United States of America **2019**; 116:3722–7.

3. Clifton LA, Skoda MW, Daulton EL, et al. Asymmetric phospholipid: lipopolysaccharide bilayers; a Gram-negative bacterial outer membrane mimic. Journal of the Royal Society, Interface / the Royal Society **2013**; 10:20130810.

4. James M, Nelson A, Holt SA, Saerbeck T, Hamilton WA, klose F. The multipurpose time-of-flight neutron reflectometer “Platypus” at Australia's OPAL reactor. Nuclear Instruments and Methods in Physics Research Section A **2011**; 632:112–23.

5. Le Brun AP, Huang TY, Pullen S, Nelson ARJ, Spedding J, Holt SA. Spatz: the time-of-flight neutron reflectometer with vertical sample geometry at the OPAL research reactor. J Appl Crystallogr **2023**; 56:18–25.

6. Nelson A. Co-refinement of multiple-contrast neutron/X-ray reflectivity data usingMOTOFIT. Journal of Applied Crystallography **2006**; 39:273–6.

7. Nelson ARJ, Prescott SW. refnx: neutron and X-ray reflectometry analysis in Python. J Appl Crystallogr **2019**; 52:193–200.

8. Holt SA, Oliver TE, Nelson ARJ. Using refnx to Model Neutron Reflectometry Data from Phospholipid Bilayers. Methods in molecular biology **2022**; 2402:179–97.
